# Alanine Scanning Mutagenesis of the DRYxxI Motif and Intracellular Loop 2 of Human Melanocortin-4 Receptor

**DOI:** 10.3390/ijms21207611

**Published:** 2020-10-15

**Authors:** Li-Kun Yang, Ya-Xiong Tao

**Affiliations:** Department of Anatomy, Physiology and Pharmacology, Auburn University College of Veterinary Medicine, Auburn, AL 36849, USA; lzy0020@auburn.edu

**Keywords:** melanocortin-4 receptor, alanine-scanning mutagenesis, binding, signaling, constitutive activity

## Abstract

The melanocortin-4 receptor (MC4R) is a member of the G-protein-coupled receptor (GPCR) superfamily, which has been extensively studied in obesity pathogenesis due to its critical role in regulating energy homeostasis. Both the Gs-cAMP and ERK1/2 cascades are known as important intracellular signaling pathways initiated by the MC4R. The DRYxxI motif at the end of transmembrane domain 3 and the intracellular loop 2 (ICL2) are thought to be crucial for receptor function in several GPCRs. To study the functions of this domain in MC4R, we performed alanine-scanning mutagenesis on seventeen residues. We showed that one residue was critical for receptor cell surface expression. Eight residues were important for ligand binding. Mutations of three residues impaired Gs-cAMP signaling without changing the binding properties. Investigation on constitutive activities of all the mutants in the cAMP pathway revealed that six residues were involved in constraining the receptor in inactive states and five residues were important for receptor activation in the absence of an agonist. In addition, mutations of four residues impaired the ligand-stimulated ERK1/2 signaling pathway without affecting the binding properties. We also showed that some mutants were biased to the Gs-cAMP or ERK1/2 signaling pathway. In summary, we demonstrated that the DRYxxI motif and ICL2 were important for MC4R function.

## 1. Introduction

Obesity, associated with several adverse health conditions, such as type 2 diabetes mellitus, hypertension, cardiovascular disease, and certain types of cancer [1], has become a critical health issue worldwide. Obesity is commonly caused by imbalanced energy intake and expenditure. Many genetic factors are involved in the development of obesity [2]. The melanocortin-4 receptor (MC4R), highly expressed in the central nervous system (CNS), is one of the major factors in obesity pathogenesis. MC4R is crucial in the regulation of both food intake and energy expenditure [3]. Targeted deletion of *Mc4r* in mice causes maturity-onset obesity associated with hyperphagia, hyperinsulinemia, and hyperglycemia [3]. Human genetic studies also revealed that 5.8% of subjects with severe obesity commencing at childhood have mutations in *MC4R*, demonstrating dysfunction of MC4R to be the most common cause of monogenic obesity [4].

As a member of family-A G-protein-coupled receptors (GPCRs), MC4R has seven hydrophobic transmembrane domains (TMDs) connected by several intracellular and extracellular loops (ICLs and ECLs) [5]. Activation of MC4R results in GDP/GTP exchange in the α-subunit of stimulatory G (Gs) protein. The α-subunit disassociates from βγ heterodimer and activates adenylyl cyclase (AC) to increase the intracellular cyclic AMP (cAMP) level and subsequently enhance protein kinase A (PKA) activity. This conventional Gs-cAMP signaling pathway is crucial in inducing anorexigenic effect to result in a negative energy balance. In addition, MC4R also activates ERK1/2 [6,7,8,9], one of three mitogen-activated protein kinases (MAPK) pathways. The ERK1/2 activation through MC4R has been shown to regulate energy homeostasis by inhibiting food intake [10,11]. Defects in ERK1/2 signaling may also contribute to obesity pathogenesis in MC4R mutation carriers [12]. Therefore, both the Gs-cAMP and ERK1/2 signaling pathways are related to the MC4R function of energy homeostasis.

Constitutive activation of GPCRs is characterized by signaling in the absence of agonist stimulation. The constitutive activity of MC4R is essential for maintaining normal energy homeostasis in humans. It has been suggested that defects in constitutive activity of MC4R in the cAMP pathway attenuate the tonic satiety signal resulting in dysfunctional energy balance and obesity [13,14]. A recent study suggested that constitutive and agonist-induced MC4R activations differentially modulate signal to impact on distinct subtypes of voltage-gated calcium channels [15]. The constitutive activity of MC4R can affect the specific channels controlling transcriptional activity coupled to depolarization and neurotransmitter release [15]. Moreover, MC4R can also be constitutively active in the ERK1/2 pathway [9], suggesting that the constitutive activation of the ERK1/2 pathway may be involved in maintaining normal energy homeostasis.

The interactions involving highly conserved residues at the cytoplasmic surface are crucial for signaling properties in GPCRs. Crystal structural studies in several family-A GPCRs reveal that the DRYxxI motif in the end of TMD3 is of importance for receptor function. Conversed amino acids in the DRYxxI motif and TMD6 form polar interactions (commonly termed as ‘ionic lock’), bridging two transmembrane domains to stabilize the receptor in an inactive conformation [16,17]. Once the receptor binds to ligands, this ‘ionic lock’ is broken and the new interaction forms between DRYxxI motif and TMD5, triggering the receptor into an active conformation [17,18]. Therefore, the DRYxxI motif is critical in constraining the receptor in inactive conformation. The ICL2, linking TMD3 and TMD4, serves as a platform for hydrogen-bonding interaction between a conserved tyrosine on the ICL2 and DRYxxI motifs [17]. ICL2 participates in G-protein coupling and β-arrestin binding, indicating that this loop is significant in receptor activation and desensitization [19].

One of our previous studies based on alanine-scanning mutagenesis has shown that the DRYxxI motif and ICL2 are critical for MC3R function [20]. However, systematic study of this domain in MC4R is still lacking. In order to enhance the understanding of the structure–function relationship of MC4R, we generated seventeen mutants in total using alanine-scanning mutagenesis to investigate the functional roles of residues in this domain. Cell surface expression, ligand binding and signaling properties of the Gs-cAMP and ERK1/2 pathways of these seventeen mutants were investigated in the present study.

## 2. Results

**Figure 1:**
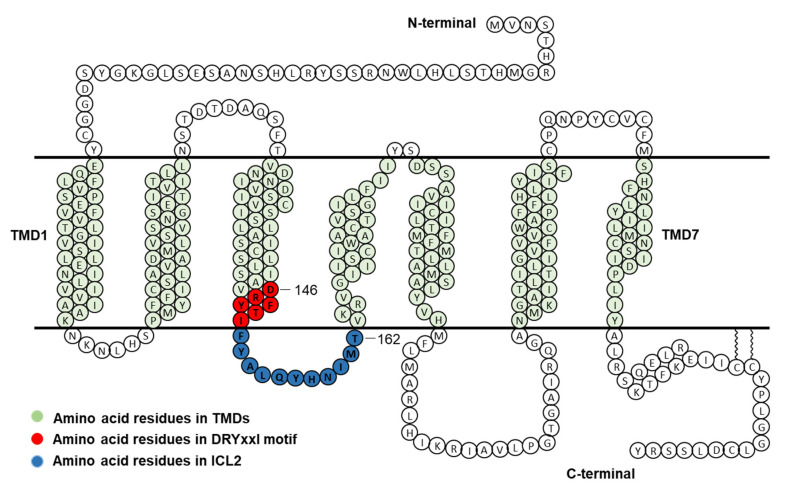
Schematic model of human MC4R. Amino acid residues in DRYxxI motif and ICL2 are indicated in red and blue circles, respectively. TMD, transmembrane domain; ICL2, intracellular loop 2.

To investigate the function of each residue of DRYxxI motif and ICL2 of human MC4R (Figure 1), alanine-scanning mutagenesis was performed to mutate each residue to alanine or alanine to glycine. A total of seventeen mutant MC4Rs were generated. NDP-MSH, a superpotent analog of endogenous agonist α-MSH [21], was used in the present study.

### 2.1. Cell Surface Expression of the Mutant MC4Rs

**Figure 2:**
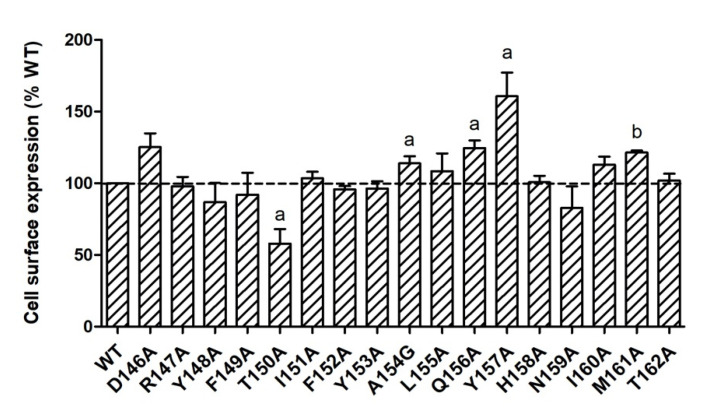
Cell surface expression of WT and mutant MC4Rs by flow cytometry. HEK293T cells were transiently transfected with WT and mutant MC4Rs. Fluorescence of Alexa Fluor 488 at non-permeabilized cells was measured. Results are expressed as % cell surface expression level of WT MC4R. Values are mean ± standard error of the mean (S.E.M.) of at least three independent experiments. The significant difference between WT and mutant: a, *p* < 0.05; b, *p* < 0.001.

It is known that GPCR mutations may impact receptors’ cell surface expression with defective protein synthesis or failure to pass through the quality control, especially in endoplasmic reticulum [22]. To quantitate the cell surface expression of mutant MC4Rs, flow cytometry technique was used in the present study. As shown in Figure 2, T150A had significantly lower cell surface expression (57.98 ± 10.03% of WT). Four mutants (A154G, Q156A, Y157A, and M161A) had slightly increased cell surface expression with statistical significance. All the other mutants were expressed at similar levels at the cell surface as WT MC4R.

### 2.2. Ligand-Binding Properties of the Mutant MC4Rs

**Figure 3:**
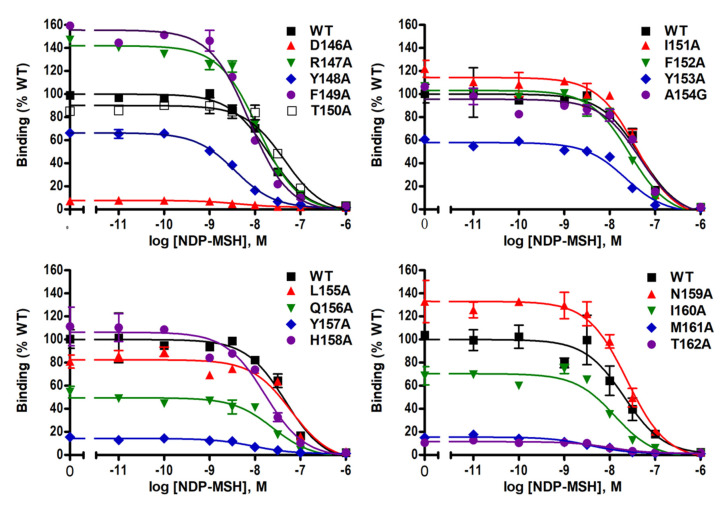
NDP-MSH binding properties of WT and mutant MC4Rs. HEK293T cells were transiently transfected with WT and mutant MC4Rs. Intact cell surface binding was measured by competitive inhibition of radiolabeled (iodine-125) NDP-MSH ([^125^I]-NDP–MSH) with different concentrations of unlabeled NDP–MSH. Results are expressed as % of WT maximal binding ± S.E.M. of duplicate measurements within one experiment. Curves are representative of three independent experiments.

Ligand-binding properties of the mutant MC4Rs were determined using unlabeled NDP-MSH to displace radiolabeled NDP-MSH. Figure 3 shows representative results and data from three independent experiments are summarized in Table 1. Of the mutant MC4Rs, D146A, Y148A, M160A, and M161A, had significantly decreased IC_50_s compared with the WT MC4R, indicating that these four mutants had increased affinities for the ligand. However, T150A had significantly increased IC_50_ (decreased affinity) compared to the WT MC4R. In addition, seven mutants (D146A, Y148A, Y153A, Q156A, Y157A, M161A, and T162A) had significantly decreased maximal binding compared to the WT MC4R whereas two mutants (F149A and N159A) exhibited increased maximal binding with statistical significance (Table 1 and Figure 3).

### 2.3. Signaling Properties of the Mutant MC4Rs in the cAMP Pathway

**Figure 4:**
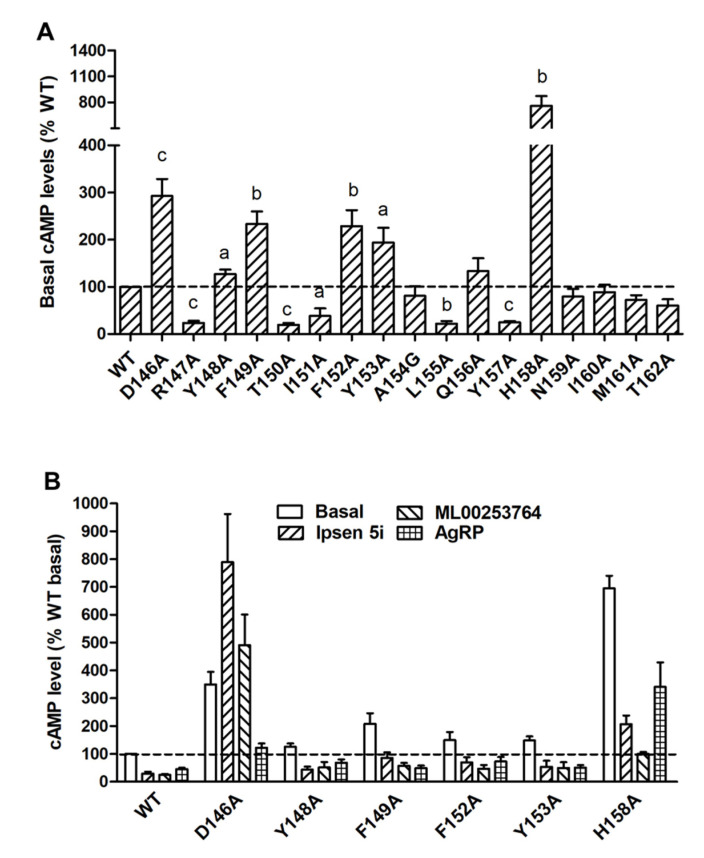
Constitutive activity of WT and mutant MC4Rs in the cAMP pathway. (**A**) HEK293T cells were transiently transfected with WT and mutant MC4Rs, and intracellular cAMP levels were measured by RIA without NDP-MSH stimulation. Results are expressed as % of basal WT cAMP level ± S.E.M. of at least three independent experiments. The basal cAMP level of WT MC4R was 41.45 ± 4.38 pmol/10^6^ cells. The significant difference between WT and mutant: a, *p* < 0.05; b, *p* < 0.01; c, *p* < 0.001. (**B**) Partial inverse agonism of Ipsen 5i, ML00253764, and AgRP (83-132) on WT and six mutants with high basal activities. HEK293T cells were transiently transfected with WT and six mutant MC4Rs. Cells were treated with 10^−6^ M Ipsen 5i, 10^−5^ M ML00253764, or 10^−8^ M AgRP (83-132), and intracellular cAMP levels were measured by RIA. Significant differences are observed between all control and treatment groups (*p* < 0.05).

To investigate both constitutive and ligand-induced signaling properties of MC4R mutants in the cAMP pathway, HEK293T cells expressing WT or mutant MC4Rs were stimulated without or with different concentrations of NDP-MSH. Five mutants (R147A, T150A, I151A, L155A, and Y157A) had significantly decreased basal cAMP levels compared to WT MC4R (Table 1 and Figure 4A). The basal cAMP production of T150A, which had reduced cell surface expression, was only 19.34 ± 4.05% of WT. However, six mutants (D146A, Y148A, F149A, F152A, Y153A, and H158A) displayed increased basal cAMP levels compared with WT (Table 1 and Figure 4A).

To further confirm that these mutants were indeed constitutively active, three inverse agonists, including the endogenous ligand AgRP (83-132) and two small molecule ligands, Ipsen 5i and ML00253764, were used to treat the cells expressing these six mutants. As shown in Figure 4B, AgRP (83-132) (10^−8^ M) could decrease the basal cAMP levels of WT and all six mutants with the inhibition ranging from 36 to 63%. The basal cAMP levels of WT and five mutants (Y148A, F149A, F152A, Y153A, and H158A) could be reduced by treatment of Ipsen 5i (10^−6^ M) or ML00253764 (10^−5^ M), with the inhibition ranging from 60 to 86%. In contrast, D146A showed activation rather than inhibition after treatment of these two small molecules.

**Figure 5:**
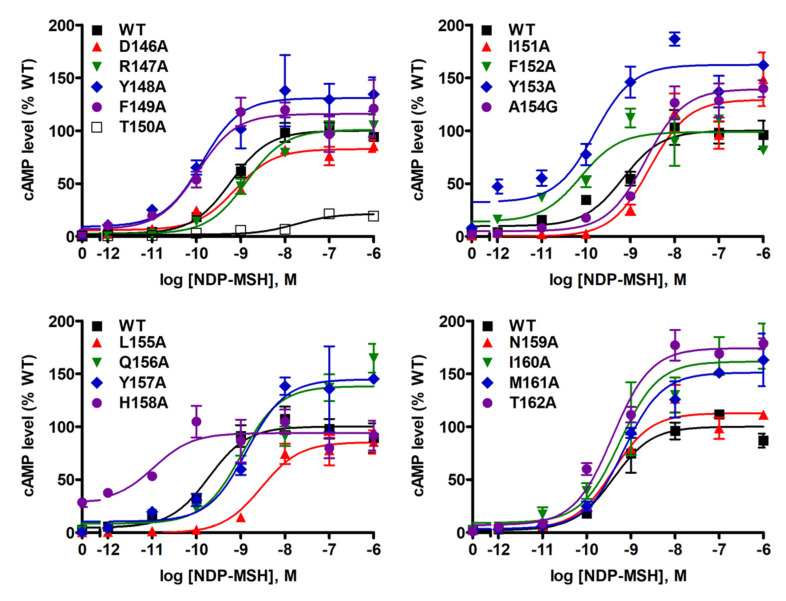
NDP-MSH-stimulated cAMP signaling properties of WT and mutant MC4Rs. HEK293T cells were transiently transfected with WT and mutant MC4Rs, and intracellular cAMP levels were measured by RIA after stimulation with different concentrations of NDP-MSH. Results are expressed as % of WT maximal response ± S.E.M. of triplicate measurements within one experiment. Curves are representative of at least three independent experiments.

NDP-MSH-stimulated cAMP levels were also measured in WT MC4R and mutants. As shown in Table 1 and Figure 5, we found that NDP-MSH dose-dependently increased intracellular cAMP accumulation in cells transfected with WT MC4R with an EC_50_ of 0.38 ± 0.07 nM. Seven mutants (D146A, R147A, T150A, I151A, L155A, Q156A, and Y157A) were less capable of producing cAMP reflected by their significantly increased EC_50_s. Two mutants (F152A and H158A) showed decreased EC_50_s, suggesting that they could respond to NDP-MSH stimulation more potently. When maximal responses were analyzed, only one mutant, T150A, was demonstrated to have remarkably decreased maximal response (17.62 ± 3.60% of WT) despite normal maximal binding. Two mutants, M161A and T162A, which had very low maximal binding, showed significantly, although very slightly, increased maximal response.

### 2.4. Signaling Properties of the Mutant MC4Rs in the ERK1/2 Pathway

**Figure 6:**
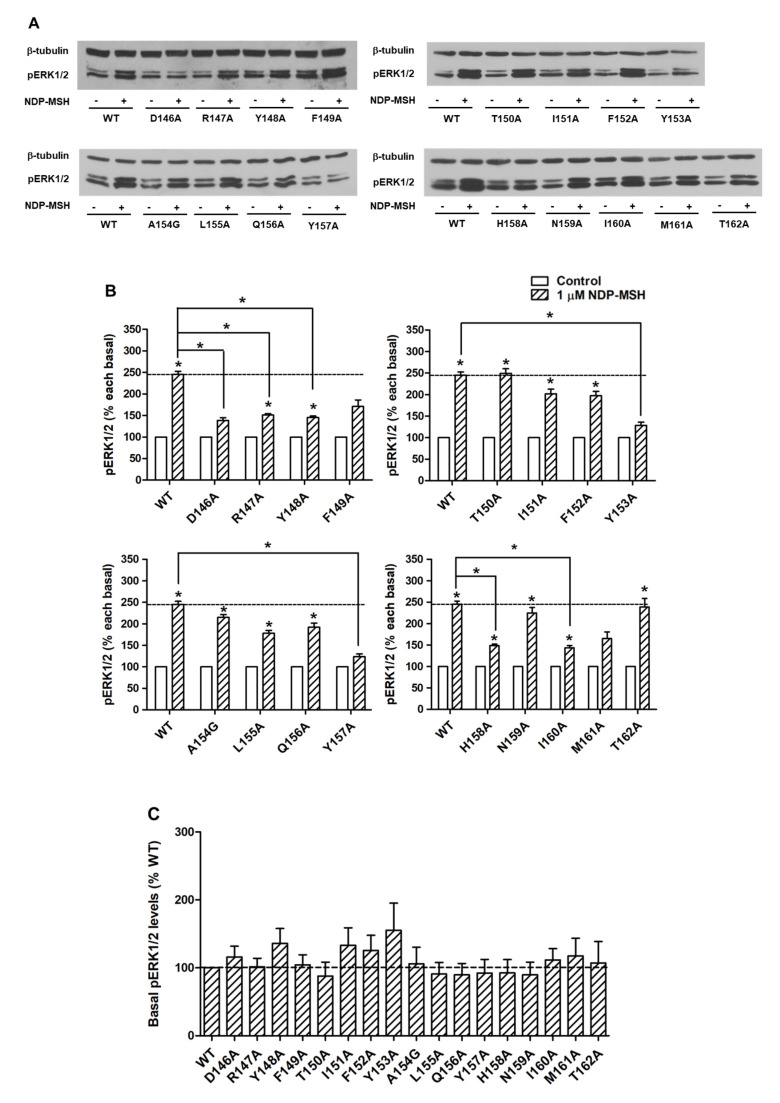
The ERK1/2 signaling properties of WT and mutant MC4Rs. HEK293T cells were transiently transfected with WT and mutant MC4Rs and pERK1/2 levels were measured by western blot without or with stimulation of 10^−6^ M NDP-MSH for 5 min. (**A**) Western blot analysis was performed using antibody against pERK1/2 and β-tubulin as control. (**B**) Data of the pERK1/2 levels of the WT and mutant MC4Rs after NDP-MSH stimulation are expressed as % of each basal from at least five independent experiments. * indicates significant difference from basal pERK1/2 level or from the stimulation of the WT MC4R (*p* < 0.05). (**C**) Data of the basal pERK1/2 levels are mean *±* S.E.M. of at least five independent experiments.

To investigate the signaling properties of mutant MC4Rs on the ERK1/2 pathway, pERK1/2 levels were measured by western blot. Our data showed that all alanine mutants did not exhibit statistically significant alterations on basal pERK1/2 levels relative to WT basal pERK1/2 level (Figure 6A,C). WT MC4R could respond to NDP-MSH stimulation in the ERK1/2 pathway with more than two-fold increased pERK1/2 level. The mutants also had significantly increased pERK1/2 levels in response to NDP-MSH except for five mutants (D146A, F149A, Y153A, Y157A, and M161A) that were not able to respond to NDP-MSH stimulation (Figure 6A,B). Although the pERK1/2 levels of four mutants (R147A, Y148A, H158A, and I160A) were increased significantly upon NDP-MSH stimulation, the fold changes were significantly lower than those of the WT receptor (Figure 6B).

## 3. Discussion

In the present study, we performed a systematic investigation on the seventeen residues of the DRYxxI motif and ICL2 of MC4R using alanine-scanning mutagenesis. The flow cytometry experiment was performed to quantitate the mutant receptor expression on cell membranes. All the mutant MC4Rs had normal or higher cell surface expression compared with the WT MC4R except for one mutant T150A with ~60% cell surface expression level of the WT receptor, suggesting defective synthesis or intracellular retention of the T150A mutant (Figure 2). One naturally occurring mutation, T150I, also has defective cell surface expression [23]. We speculated that T150 might be a critical site for cell surface expression of MC4R. Impaired cell surface expression is the most common defect of naturally occurring mutations in the MC4R [24].

Competitive binding assay revealed that alanine mutations of eight residues (D146, Y148, T150, Y153, Q156, Y157, M161, and T162) impaired ligand binding with either increased IC_50_s or decreased maximal binding (Table 1 and Figure 3). D146A, M161A, and T162A caused severely reduced maximal binding (Table 1 and Figure 3), which was similar to our previous finding that alanine mutants of corresponding residues in MC3R (D178, M193, and T194) exhibited undetectable binding [20], suggesting that these three residues were important for ligand binding in both melanocortin receptors. Residues in the DRYxxI motif and ICL2 are located at the transmembrane and cytoplasmic region (Figure 1). Although these residues are remote from the binding pocket formed by residues located on the extracellular side of the MC4R [25,26], and likely not directly involved in receptor-ligand interaction, these mutations might induce conformational change that indirectly modulate the receptor-ligand interaction. Similar observations were made in other GPCRs as well as the MC3R and MC4R [20,27,28,29].

Constitutive activities of GPCRs have been suggested to play a significant role in physiology and pathophysiology [30,31,32]. Constitutive activity is also a prominent feature of MC4R (whereas the related human MC3R has minimal constitutive activity [33]). Part of the MC4R N-terminus, the “HLWNRS” sequence between position 14 and 19, embedded between the TMDs and ECLs, might serve as an intramolecular tethered agonist to generate a high basal activity [26]. Decreased constitutive activity in mutant MC4Rs is suggested to be one cause of obesity [13]. Some naturally occurring *MC4R* mutations were indeed found to decrease the constitutive activity [13,34,35,36]. Paradoxically, six mutants were shown to increase constitutive activity [37,38,39]. It remains unknown whether mutations resulting in enhanced constitutive activation of receptors are the cause of obesity (reviewed in [14]); we did not observe any defect in ERK1/2 signaling in these mutants [40].

In the cAMP assay, we found that eleven alanine mutants exhibited altered basal cAMP levels (Table 1 and Figure 4A). Mutations on the corresponding sites might shift the equilibrium between inactive and active states, resulting in either enhanced or reduced constitutive activation. Five mutant MC4Rs (R147A, T150A, I151A, L155A, and Y157A) exhibited decreased basal cAMP levels, implying that these residues were important for basal signaling. Six mutants (D146A, Y148A, F149A, F152A, Y153A, and H158A) had increased basal cAMP levels that could be partially inhibited by Ipsen 5i, ML00253764, or AgRP (83–132), except that Ipsen 5i and ML00253764 failed to decrease the basal cAMP level of D146A (Figure 4B). Our results indicated that these six mutants were indeed constitutively active mutants. The absence of inverse agonistic effects of Ipsen 5i and ML00253764 on D146A might be considered as the consequence of specific conformation of the mutant receptor-compound complex. In this complex, the receptor may be less likely to adopt inactive conformation due to alanine mutation on D146. In contrast, D146A tended to have active conformation to trigger G-protein activation, as evidenced by increased cAMP production. It is also possible that D146 is the residue or one of the residues critical for contacting Ipsen 5i and ML00253764. In fish MC4Rs, Ipsen 5i also works as an allosteric agonist [41,42]. In one previous study, an L133M mutation of MC4R also converts an antagonistic effect of SHU9199 to an agonistic effect [43]. One very recent study confirmed that there is an apparent interaction between L133 and the unnatural amino acid Nal^4^ of SHU9119 based on MC4R crystal structure [25].

It has been suggested that the highly conserved DRYxxI motif can interact with residues on TMD6 to regulate the receptor conformational switch between inactive and active states in family-A GPCRs [44,45,46,47]. The crystal structure of the inactive state of rhodopsin indicates that the basic arginine (R^3.50^) forms double salt bridges with neighboring aspartic acid (D^3.49^) and glutamic acid (E^6.30^) on TMD6 (Ballesteros and Weinstein numbering scheme [48]) to constrain receptors in the ground state [49]. Mutations on D^3.49^ in several GPCRs, such as rhodopsin, α_1B_-adrenergic receptor (AR), vasopressin type II receptor, β_2_-AR, and μ-opioid receptor, frequently induce constitutive activation [50]. Our results demonstrated that alanine substitution on D146^3.49^ of MC4R resulted in an approximately three-fold increase of basal cAMP level (Table 1 and Figure 4A), indicating the crucial role of D146^3.49^ of MC4R in constraining a receptor in its ground state. One MC4R naturally occurring mutant, D146N^3.49^, has also been shown to exhibit increased constitutive activity [36]. However, alanine mutation on R147^3.50^ of MC4R caused a decreased basal cAMP level (Table 1 and Figure 4A), implying that R147^3.50^ might be important in contacting Gαs to stabilize the active status of the receptor-G protein complex as supported by previous findings [51,52], but might not form a salt bridge with the residue on TMD6 (N240^6.30^) to constrain receptors in the ground state [8].

T150^3.53^ is also highly conserved among rhodopsin-like GPCRs. Our data showed that alanine mutations on this residue could dramatically decrease the constitutive activity (Table 1 and Figure 4A). The reduced constitutive activity of T150A should be considered in the context of its decreased cell surface expression. Among the residues important for MC4R constitutive activation in ICL2, alanine mutation on H158 caused an almost eight-fold increase in constitutive activity. Given that one naturally occurring mutant H158R has more than a six-fold increase in constitutive activity compared with WT MC4R [53], we consider that H158 might also be an important residue for constraining receptors in inactive conformation.

Seven mutants, D146A, R147A, T150A, I151A, L155A, Q156A, and Y157A, had impaired Gs-cAMP signaling upon NDP-MSH stimulation (Table 1 and Figure 5). The signaling deficiencies of three mutants (D146A, Q156A, and Y157A) were likely due to their impaired ligand-binding properties. Among other signaling defective mutants, we found that T150A had severely reduced maximal response apart from increased EC_50_ (Table 1 and Figure 5). The defective signaling properties of T150A might be caused by decreased cell surface expression (Figure 2) and binding affinity (Table 1). Alanine mutations on the corresponding position 3.53 in MC3R [20], follicle-stimulating hormone receptor [54], and angiotensin II type 1 receptor [55], were also reported to affect receptor signaling.

Alanine mutation on I151^3.54^, which is distal to the DRYxxI motif, impaired Gs-cAMP signaling (reduced constitutive activity and increased EC_50_) without changing ligand-binding properties, suggesting that this residue was also critical in signal transduction. Several previous studies have demonstrated that residue 3.54 is involved in receptor-G protein coupling. Several mutations on this residue of MC3R, including I183A^3.54^, I183N^3.54^, and I183R^3.54^, cause profound loss of agonist-stimulated cAMP production [20,56]. One MC4R mutant, I151N^3.54^, also displayed no detectable agonist-stimulated cAMP production despite normal ligand binding [56].

L155^3.58^ is a highly conserved residue in rhodopsin-like GPCRs and was reported to be critical for G-protein coupling. Impaired receptor activation caused by mutations on the corresponding position has been shown in other GPCRs, such as MC3R [20], β_2_-AR [52], and G-protein coupled receptor 54 [57]. The defective signaling properties of L155A^3.58^ also suggested that the corresponding residue was also important in MC4R activation.

It should be noted that although 4 mutants, D146A, Y157A, M161A, and T162A, had severely impaired maximal binding with ligands (less than 20% of WT MC4R maximal binding), they exhibited comparable cAMP production as WT MC4R upon NDP-MSH stimulation (Table 1, Figure 3 and Figure 5). The presence of spare receptors in a heterologous expression system might be an explanation [34,58]. Similar results were also observed in some naturally occurring MC4R mutants such as S58C and I102T [59], as well as some lab-generated alanine mutants [9].

The activation of ERK1/2 is a significant signaling cascade triggered by MC4R, which is thought to be involved in the regulation of energy homeostasis by mediating inhibition of food intake [10,11]. Five alanine mutants, including D146A, R149A, Y153A, Y157A, and M161A, could not respond to NDP-MSH stimulation in the ERK1/2 pathway (Figure 6A,B). In our previous study of the DRYxxI motif and ICL2 of MC3R, two mutant MC3Rs, Y185A and M193A (corresponding to Y153 and M161 in MC4R), are also unable to induce ERK1/2 signaling with NDP-MSH stimulation [20]. This indicated that the two residues were important for ERK1/2 signaling in both melanocortin receptors. Four alanine mutants, including R147A, Y148A, H158A, and I160A, also exhibited defective ERK1/2 activation since the efficacies of NDP-MSH in these mutants were lower compared to the WT receptor, although they could respond to stimulation significantly (Figure 6B).

Biased agonism, including biased mutants and ligands, has been proposed in MC4R by several groups [12,40,60,61,62,63] (reviewed in [64,65]). Previous studies in our lab have identified many biased MC4R mutants which trigger biased signaling between Gs-cAMP and ERK1/2 pathways [8,12,40]. Our present study also investigated biased signaling of mutant MC4Rs. With the stimulation of NDP-MSH, one mutant T150A could induce biased ERK1/2 activation, whereas five mutants (Y148A, F149A, H158A, I160A, and M161A) exhibited biased Gs-cAMP signaling (Table 1, Figure 5 and Figure 6).

The regulation of metal ions (e.g., Na^+^, Zn^2+^, Cu^2+^, and Ca^2+^) on MC4R function, such as ligand binding, constitutive activity, and ligand-induced activity, has attracted much interest in recent years. For example, Na^+^ can allosterically modulate MC4R activity [66] and D90^2.50^ residue, as revealed by NMR structure, participates in maintaining the functional conformation of MC4R by Na^+^ interaction [67]. Zn^2+^ and Cu^2+^ are also shown to allosterically modulate constitutive activity of MC4R in the cAMP pathway [68]. In the crystal structure of MC4R, Ca^2+^ was identified to be a cofactor for ligand binding at orthosteric sites and regulate the affinity and potency of α-MSH, but not AgRP, in a selective manner [25]. Although our present study did not investigate receptor functionalities in the context of metal ion regulation, it is of particular interest for future studies to take this into consideration and explore the structure–function relationship of MC4R under physiological ion conditions.

**Figure 7:**
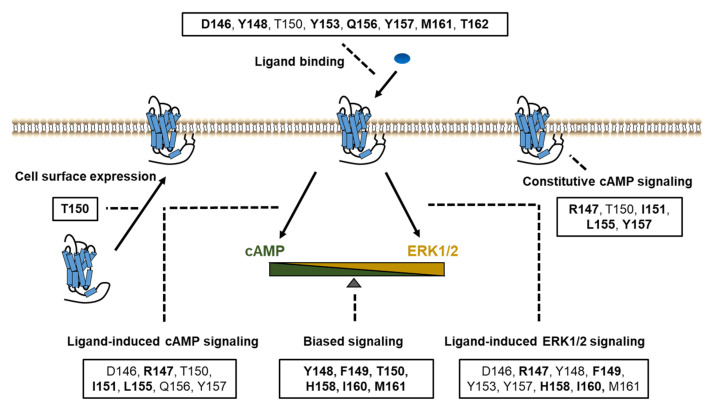
Summary of the functions of the DRYxxI motif and ICL2. Impaired cell surface expression, ligand binding, and/or signaling pathways were due to alanine mutations of the residues shown in boxes. The residues indicated in bold font, are thought as critical residues for the relevant function of receptors, on which alanine mutations primarily cause defective activities.

In summary, based on systematic study of the DRYxxI motif and ICL2 of MC4R, we have identified the residues that were crucial for ligand binding and signaling in Gs-cAMP and ERK1/2 pathways (Figure 7). Biased signaling was also investigated in our present study, which expands our understanding of the MC4R intracellular signaling. The structure–function relationship of MC4R data presented herein contributes to a better understanding of MC4R pharmacology, which might be useful for rationally designing agonists and antagonists for treatment of MC4R-related diseases.

## 4. Materials and Methods

### 4.1. Materials

Human embryonic kidney 293T (HEK293T) cells were purchased from American Type Culture Collection (Manassas, VA, USA). [Nle^4^, D-Phe^7^]-α-melanocyte-stimulating hormone (NDP-MSH) and agouti-related peptide (AgRP) (83-132) were purchased from Peptides International (Louisville, KY, USA). Ipsen 5i [69] and ML00253764 [35] were synthesized by Enzo Life Sciences, Inc. (Plymouth Meeting, PA, USA). [^125^I]-NDP-MSH and [^125^I]-cAMP were iodinated using the chloramine-T method as described previously [9,70]. The N-terminal c-myc-tagged WT MC4R subcloned into pcDNA3.1 vector (Invitrogen, Carlsbad, CA, USA) was generated as previous described [59].

### 4.2. Site-Directed Mutagenesis

Mutant MC4Rs were generated from the WT receptor by QuikChange site-directed mutagenesis kit (Stratagene, La Jolla, CA, USA) using the primers listed in Table 2. The sequences were verified by direct nucleotide sequencing by the DNA Sequencing Facility of University of Chicago Cancer Research Center (Chicago, IL, USA).

### 4.3. Cell Culture and Transfection

HEK293T cells were cultured in Dulbecco’s Modified Eagle’s Medium (DMEM) (Invitrogen) containing 10% newborn calf serum (PAA Laboratories Inc., Etobicoke, ON, Canada), 10 mM HEPES, 100 units/mL penicillin, 100 μg/mL streptomycin, 50 μg/mL of gentamicin, and 0.25 μg/mL amphotericin B, at 37 °C and in 5% CO_2_ humidified atmosphere. For flow cytometry, ligand binding, and cAMP signaling studies, cells were seeded into gelatin-coated six-well plates (Corning, NY, USA) and were transfected with WT or mutant MC4Rs at approximately 70% confluence using the calcium phosphate precipitation method as described previously [71]. For western blot, cells were plated into gelatin-coated 100 mm dishes (Corning) and transfected using same method.

### 4.4. Quantification of Receptor Cell Surface Expression by Flow Cytometry

HEK293T cells were transfected as described above. Approximately 48 h after transfection, six-well plates were placed on ice, and cells were washed once with cold PBS for immunohistochemistry (PBS-IH) [59], detached with PBS-IH, and centrifuged at 1500 rpm for 5 min. Then, cells were fixed with 4% paraformaldehyde in PBS-IH for 15 min. After blocking with PBS-IH containing 5% bovine serum albumin (BSA, EMD Millipore Corporation, Burlington, MA, USA) for 1 h, cells were incubated for another 1 h, with the primary antibody 9E10 monoclonal anti-myc antibody (Developmental Studies Hybridoma Bank at the University of Iowa, Iowa City, IA, USA) 1:40 diluted in PBS-IH containing 0.5% BSA (PBS-IH/BSA). After incubation, cells were washed once with PBS-IH/BSA, and incubated for 1 h, with the secondary antibody Alexa Fluor 488-conjugated goat anti-mouse IgG (Invitrogen) 1:1500 diluted in PBS-IH/BSA. After incubation, cells were washed once with PBS-IH/BSA, filtered to obtain single cell suspension, and assayed using a C6 Accuri Cytometer (Accuri Cytometers, Ann Arbor, MI, USA). Cells transfected with pcDNA3.1 were used as control for background staining. The expression levels of mutant MC4Rs were calculated by the following formula: (mutant-pcDNA3.1)/(WT-pcDNA3.1) × 100% [72].

### 4.5. Competitive Ligand Binding Assay

HEK293T cells were transfected as described above. Approximately 48 h after transfection, cells were washed with warm DMEM containing 1 mg/mL BSA. Then, cells were incubated with DMEM/BSA, ~80,000 cpm of [^125^I]-NDP-MSH, and without or with different concentrations of unlabeled NDP-MSH at 37 °C (final concentrations ranged from 10^−11^ to 10^−6^ M). One hour later, cells were washed with cold Hank’s balanced salt solution containing 1 mg/mL BSA, lysed with 100 μL 0.5 N NaOH, and collected by cotton swabs. The radioactivity of cells lysates was measured in a gamma counter (Cobra II Auto-Gamma, Packard Bioscience Co., Frankfurt, Germany).

### 4.6. cAMP Assay

HEK293T cells were transfected as described above. Approximately 48 h after transfection, cells were washed with warm DMEM/BSA and incubated with fresh DMEM/BSA containing 0.5 mM isobutylmethylxanthine (Sigma-Aldrich, St. Louis, MO, USA) at 37 °C for 30 min. Cells were then treated without or with NDP-MSH (final concentrations ranged from 10^−12^ to 10^−6^ M) for investigation of ligand-induced cAMP signaling. Cells were also treated without or with Ipsen 5i (10^−6^ M), ML00253764 (10^−5^ M), or AgRP (83-132) (10^−8^ M) for investigation of constitutive activities of some mutant MC4Rs in cAMP signaling. After 1 h incubation, cells were lysed with 0.5 M perchloric acid containing 180 μg/mL theophylline (Sigma-Aldrich) and neutralized by 0.72 M KOH/0.6 M KHCO_3_. The cAMP levels were determined by radioimmunoassay (RIA) as described previously [70].

### 4.7. Protein Preparation and Western Blot

For protein preparation, HEK293T cells were transfected as described above. Approximately 24 h after transfection, cells were starved in DMEM/BSA at 37 °C for 24 h. On the day of experiment, cells were treated without or with 10^-6^ M NDP-MSH at 37 °C for 5 min. After treatment, cells were then solubilized in lysis buffer containing phosphatase and protease inhibitors. Total protein concentrations of cell lysates were determined using the Bradford protein assay.

For western blot, 35 μg protein samples were separated on 10% SDS-PAGE gel and transferred onto PVDF membrane for immunoblotting. The PVDF membranes were blocked in 10% nonfat dry milk (containing 0.2% Tween-20) for 4 h at room temperature, and immunoblotted with rabbit anti-phosphorylated ERK1/2 (pERK1/2) antibody (Cell signaling, Beverly, MA, USA) 1:5000 and mouse anti-β-tubulin antibody (Development Studies Hybridoma Bank, University of Iowa, Iowa City, IA, USA) 1:5000 diluted in Tris-buffered saline containing Tween-20 (TBST) with 5% BSA overnight at 4 °C. On the next day, PVDF membranes were washed with TBST for 1 h and then probed with horseradish peroxidase-conjugated secondary antibodies, donkey anti-rabbit IgG 1:5000 and donkey anti-mouse IgG (both from Jackson ImmunoResearch Laboratories, West Grove, PA, USA) IgG 1:5000 diluted in 10% nonfat dry milk for 1~2 h at room temperature. Membranes were washed with TBST for 1 h and specific bands were detected with ECL reagent (Thermo Scientific, Rockford, IL, USA) and were analyzed and quantified by ImageJ Software (NIH, Bethesda, MD, USA).

### 4.8. Statistical Analysis

GraphPad Prism v.4.0 Software (San Diego, CA, USA) was used to calculate the ligand-binding parameters including IC_50_ and maximal binding, and cAMP signaling parameters including EC_50_ and maximal response. To determine the significance of differences in cell surface expression, ligand binding and cAMP signaling parameters, and pERK1/2 levels between WT and mutant MC4Rs, two-tailed student’s *t* test was performed.

## Figures and Tables

**Table 1:** The ligand-binding and signaling properties of WT and mutant MC4Rs. Values are expressed as the mean ± standard error of the mean (S.E.M.) of at least three independent experiments. The maximal response (*R*_max_) of WT MC4R was 1662.19 ± 136.63 pmol cAMP/10^6^ cells with NDP-MSH stimulation.

	NDP-MSH Binding			Basal Activity (% WT)		NDP-MSH-Stimulated cAMP			*n*	NDP-MSH-Stimulated ERK1/2
	*n*	IC_50_ (nM)	*B*_max_ (% WT)	*n*	Basal cAMP levels	*n*	EC_50_ (nM)	*R*_max_ (% WT)		
**WT**	14	19.43 ± 2.10	100 ± 0	31	100 ± 0	25	0.38 ± 0.07	100 ± 0	16	+
**D146A**	3	3.24 ± 0.70 ^a^	6.47 ± 0.97 ^b^	9	292.67 ± 35.94 ^b^	4	1.07 ± 0.18 ^a^	108.52 ± 12.25	6	–
**R147A**	3	11.86 ± 0.25	176.71 ± 38.93	4	23.62 ± 4.31 ^b^	4	1.73 ± 0.44 ^a^	121.75 ± 14.65	6	–
**Y148A**	3	4.31 ± 1.78 ^b^	60.38 ± 10.62 ^a^	9	126.90 ± 9.86 ^a^	3	0.21 ± 0.09	136.92 ± 8.17 ^a^	6	–
**F149A**	3	6.30 ± 0.83	156.37 ± 13.73 ^a^	8	233.51 ± 26.15 ^a^	3	0.24 ± 0.08	154.21 ± 31.84	5	–
**T150A**	3	40.25 ± 6.11 ^a^	95.30 ± 3.43	4	19.34 ± 4.05 ^b^	4	11.55 ± 2.38 ^b^	17.62 ± 3.60 ^b^	6	+
**I151A**	4	17.78 ± 6.96	124.47 ± 28.35	3	38.42 ± 15.77 ^a^	3	3.29 ± 0.27 ^b^	93.52 ± 24.82	5	+
**F152A**	3	17.29 ± 5.63	76.21 ± 14.64	11	228.74 ± 33.95 ^b^	5	0.06 ± 0.02 ^a^	115.47 ± 14.57	5	+
**Y153A**	3	13.02 ± 5.03	56.84 ± 4.52 ^b^	9	194.08 ± 31.05 ^b^	3	0.26 ± 0.10	152.24 ± 28.47	5	–
**A154G**	3	28.72 ± 9.72	84.83 ± 6.18	3	80.70 ± 21.17	3	0.87 ± 0.10	110.06 ± 10.78	8	+
**L155A**	3	50.80 ± 14.05	79.61 ± 8.63	4	21.77 ± 5.64 ^b^	4	6.03 ± 1.42 ^b^	100.44 ± 5.25	7	+
**Q156A**	3	15.41 ± 5.45	61.26 ± 6.10 ^b^	4	133.59 ± 27.19	4	1.76 ± 0.43 ^a^	129.25 ± 23.87	8	+
**Y157A**	3	7.43 ± 2.02	14.88 ± 2.18 ^b^	5	24.72 ± 2.89 ^b^	4	2.85 ± 0.61 ^b^	131.64 ± 18.30	7	–
**H158A**	3	10.36 ± 3.15	138.97 ± 25.34	8	761.86 ± 111.54 ^b^	3	0.03 ± 0.02 ^a^	109.75 ± 26.84	7	–
**N159A**	3	15.19 ± 5.31	119.92 ± 6.62 ^a^	4	79.16 ± 16.55	4	0.30 ± 0.07	92.29 ± 16.32	7	+
**I160A**	3	6.77 ± 2.07 ^a^	95.24 ± 16.43	3	88.84 ± 15.64	3	0.20 ± 0.18	109.91 ± 28.73	7	–
**M161A**	4	3.47 ± 1.19 ^b^	13.18 ± 2.67 ^b^	4	72.38 ± 9.95	4	0.41 ± 0.16	137.22 ± 11.70 ^a^	7	–
**T162A**	3	5.79 ± 2.35	13.73 ± 1.17 ^b^	4	60.37 ± 13.41	4	0.46 ± 0.10	145.81 ± 13.95 ^a^	8	+

^a^ significantly different from WT, *p* < 0.05; ^b^ significantly different from WT, *p* < 0.001.

**Table 2:** Forward primer sequences used for site-directed mutagenesis studies of human MC4R. The mutated codons are underlined and the mutated nucleotides are in bold.

Constructs	Forward Primer Sequences (5′→3′)
D146A	CAATTGCAGTG**G**CCAGGTACTTTAC
R147A	CAATTGCAGTGGAC**G**CGTACTTTACT
Y148A	GCAGTGGACAGG**GC**CTTTACTATCTT
F149A	CAGTGGACAGGTAC**GC**TACTATCTTC
T150A	GACAGGTACTTT**G**CTATCTTCTATG
I151A	CAGGTACTTTACT**GC**CTTCTATGCTCT
F152A	GTACTTTACTATC**GC**CTATGCTCTCCA
Y153A	CTTTACTATCTTC**GC**TGCTCTCCAGTA
A154G	CTATCTTCTATG**G**TCTCCAGTACC
L155A	CTATCTTCTATGCT**GC**CCAGTACCATA
Q156A	CTATGCTCTC**GC**GTACCATAAC
Y157A	CTATGCTCTCCAG**GC**CCATAACATTAT
H158A	GCTCTCCAGTAC**GC**TAACATTATGAC
N159A	CTCCAGTACCAT**GC**CATTATGACAG
I160A	CCAGTACCATAAC**GC**TATGACAGTTA
M161A	GTACCATAACATT**GC**GACAGTTAAGC
T162A	CATAACATTATG**G**CAGTTAAGCGGG

## Abbreviations

AC	Adenylyl cyclase
AgRP	Agouti-related peptide
CNS	Central nervous system
EC_50_	Half maximal effective concentration
ECL	Extracellular loop
ERK1/2	Extracellular signal-regulated protein kinases 1 and 2
GPCR	G protein-coupled receptor
Gs	Stimulatory G protein
HEK293T	Human embryonic kidney 293T
IC_50_	Half maximal inhibitory concentration
ICL	Intracellular loop
MAPK	Mitogen-activated protein kinase
MC3R	Melanocortin-3 receptor
MC4R	Melanocortin-4 receptor
MSH	Melanocyte-stimulating hormone
NDP-MSH	[Nle^4^, D-Phe^7^]-α-melanocyte-stimulating hormone
pERK1/2	Phosphorylated ERK1/2
PKA	Protein kinase A
TMD	Transmembrane domain
